# A brief history of visualizing membrane systems in molecular dynamics simulations

**DOI:** 10.3389/fbinf.2023.1149744

**Published:** 2023-05-05

**Authors:** R. A. Corey, M. Baaden, M. Chavent

**Affiliations:** ^1^ Department of Biochemistry, University of Oxford, Oxford, United Kingdom; ^2^ Centre Nationale de la Recherche Scientifique, Laboratoire de Biochimie Théorique, Université Paris Cité, Paris, France; ^3^ Institut de Pharmacologie et Biologie Structurale, CNRS, Université de Toulouse, Toulouse, France

**Keywords:** molecular dynamics simulation, multiscale modelling and simulation, membrane system, molecular graphics, membrane protein

## Abstract

Understanding lipid dynamics and function, from the level of single, isolated molecules to large assemblies, is more than ever an intensive area of research. The interactions of lipids with other molecules, particularly membrane proteins, are now extensively studied. With advances in the development of force fields for molecular dynamics simulations (MD) and increases in computational resources, the creation of realistic and complex membrane systems is now common. In this perspective, we will review four decades of the history of molecular dynamics simulations applied to membranes and lipids through the prism of molecular graphics.

## Introduction

Understanding lipid dynamics and function, from the level of single, isolated molecules to large assemblies, is more than ever an intensive area of research (Levental and Lyman, 2022). The interactions of lipids with other molecules, particularly membrane proteins, are now extensively studied. With advances in the development of force fields for molecular dynamics simulations (MD) and increases in computational resources, the creation of realistic and complex membrane systems is now common. This situation poses a major challenge for the analysis of these systems to make sense of what is perceived *a priori* as a sum of erratic movements and interactions.

The metaphor of the fluid mosaic, introduced 50 years ago (Singer and Nicolson, 1972) to conceptualize membrane systems, is still alive, even if the scenario we observe at the nanoscale is more like a soup of molecules. To understand how all these *ingredients* come together to give each membrane system (bacterial, eukaryotic, etc.) its typical flavor, computational biologists have developed numerous strategies over the years. In this perspective, we will discuss how advances in modeling have driven the use of more sophisticated visualizations and analyses to decipher lipid interactions and dynamics from the molecular level to large supramolecular assemblies. To this end, we will review four decades of the history of molecular dynamics simulations applied to membranes and lipids through the prism of molecular graphics. We cannot cover this area exhaustively in such a short format. Therefore, we recommend that interested readers take a look at the recent contributions on membrane modeling (Enkavi et al., 2019; Marrink et al., 2019; Muller et al., 2019) and molecular visualization in general (Martinez et al., 2019; Martinez et al., 2020a).

## The eighties: less graphics and more analysis

**FIGURE 1:**
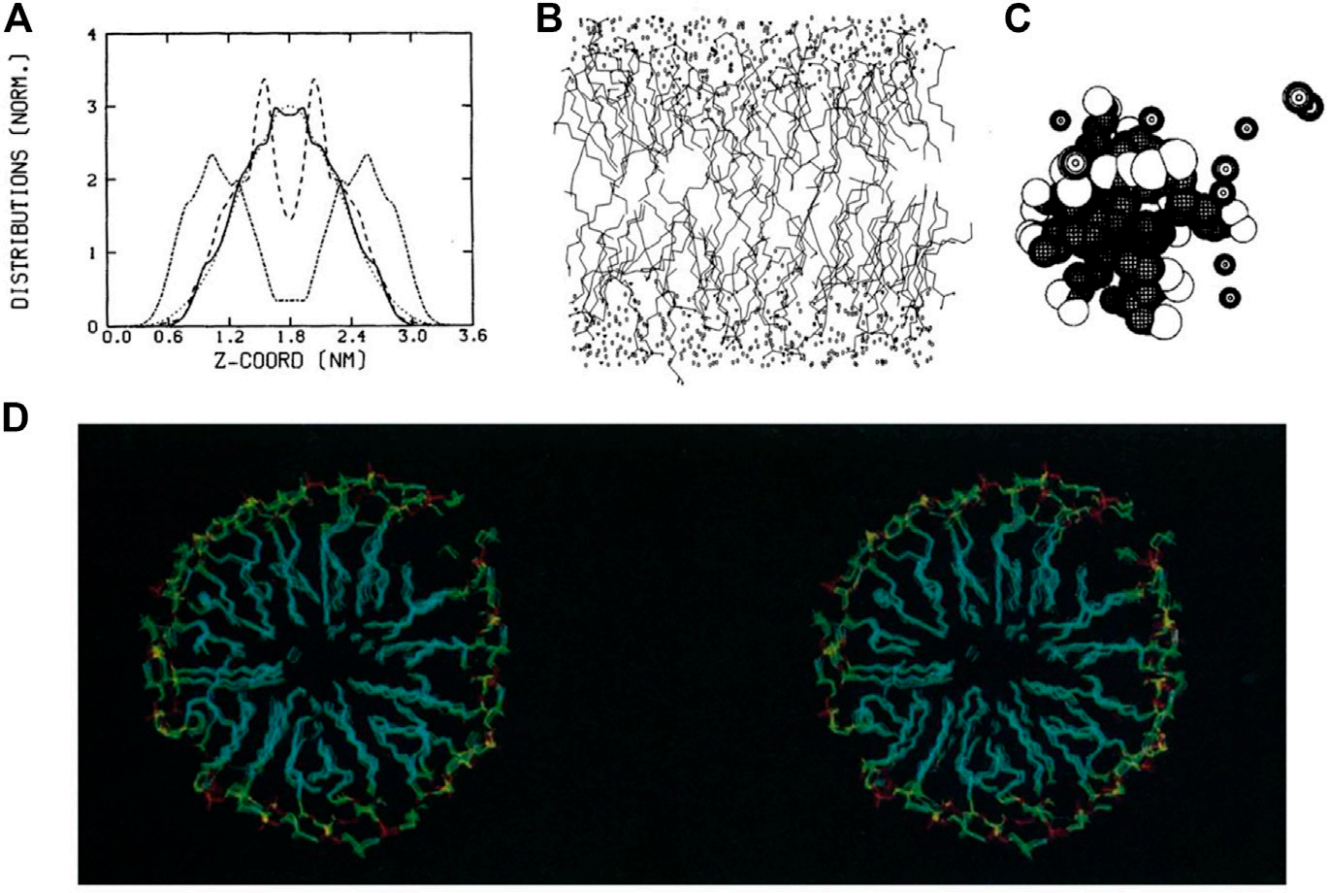
Examples of representations and analyses developed in the 1980s to decipher lipid assemblies. **(A)** Density analysis showing the z-organisation of lipids as a bilayer (Egberts and Berendsen, 1988). **(B)** Line representations of lipids, water and ion molecules (Egberts and Berendsen, 1988). **(C)** Sphere rendering of a micelle (Jönsson et al., 1986). **(D)** Stereoscopic view of a lipid micelle (Wendoloski et al., 1989). Reprinted with permission from AAAS.

In the 1980s, molecular dynamics simulations were limited in both simulation time and system size (Egberts and Berendsen, 1988). With the use of atomistic forcefields (Gunsteren and Berendsen, 1990), systems were simulated for only a short period of time, typically picoseconds. Thus, analyses and visualizations of lipids dynamics were relatively limited. Nevertheless, analyses being developed and applied at that time are still in use today, such as order parameters for lipid tails, radial distribution function, calculation of lateral diffusion and distribution of atoms along the membrane-normal *z*-axis (Figure 1A). Conversely, the 3D graphics were quite simple and only served to provide an overview of the simulated system. The rendering consisted mainly of black and white lines (Egberts and Berendsen, 1988) or spheres (Brickmann, 1984) (Figures 1B, C), which leaves interpretation difficult. Regarding dynamics, the analysis focused mainly on the internal movements of lipids, since the simulation time didn’t allow for major membrane changes.

Membrane proteins, more specifically peptides, were studied to understand how they can fold and remain in an apolar environment (Edholm and Jähnig, 1988). Again, the representation of these models was based on lines connecting atoms. In some works, especially for the representation of proteins, the authors proposed side-by-side images of the protein structures in a so-called stereoscopic view, which the reader can use with appropriate stereo glasses or a specific device to grasp the spatial context of these 3D structures [see, e.g. (Sessions et al., 1989)]. At the end of this decade, stereoscopic views with colored images began to be used to visualize lipid assemblies such as micelles (Wendoloski et al., 1989) (Figure 1D).

Over the next decade, more advanced rendering techniques were developed and hardware was improved to enable numerous new representations that mixed different molecular metaphors to characterize more complex systems.

## 1990–2005: The advent of molecular viewers to render more complex scenes

Even though interactivity was already possible with the early pioneering molecular visualization programs (Olson, 2018), the development of molecular viewers (O’Donoghue et al., 2010), as we know them today, was booming in the period 1990–2005, when several such programs were developed, including VMD (Humphrey et al., 1996), PyMOL (DeLano, 2002), and Chimera (Pettersen et al., 2004), all of which are still in use today. This effort was closely related to the development of new versions of MD programs such as Gromacs 3.0 (Lindahl et al., 2001) or NAMD2 (Kalé et al., 1999), which could simulate a wider variety of systems over a longer period of time. Even though interactivity gradually became the norm during this decade, the non-interactive use of raster image programs based on advanced shading algorithms was still very attractive (Merritt and Murphy, 1994).

For membrane systems, force fields developments (Mackerell, 2004) enabled the modeling of different types of lipids (Essex et al., 1994; Höltje et al., 2001; Lins and Straatsma, 2001; Feller et al., 2003; Hofsäß et al., 2003; Pandit et al., 2004) and their interactions with small molecules (Bassolino-Klimas et al., 1993), or peptides and proteins (Woolf and Roux, 1994; Damodaran et al., 1995; Edholm et al., 1995; Zhou and Schulten, 1996; Tieleman and Berendsen, 1998). These features allowed the study of more complex systems (Saam et al., 2002; Tang and Xu, 2002). This increasing complexity, in turn, required the combination of different visualizations (such as line, licorice, Van der Waals, or secondary structure representations) to comprehensively represent both the entire scene and precise details to understand the dynamic behavior of molecules. Several use cases required more sophisticated representation.

**FIGURE 2:**
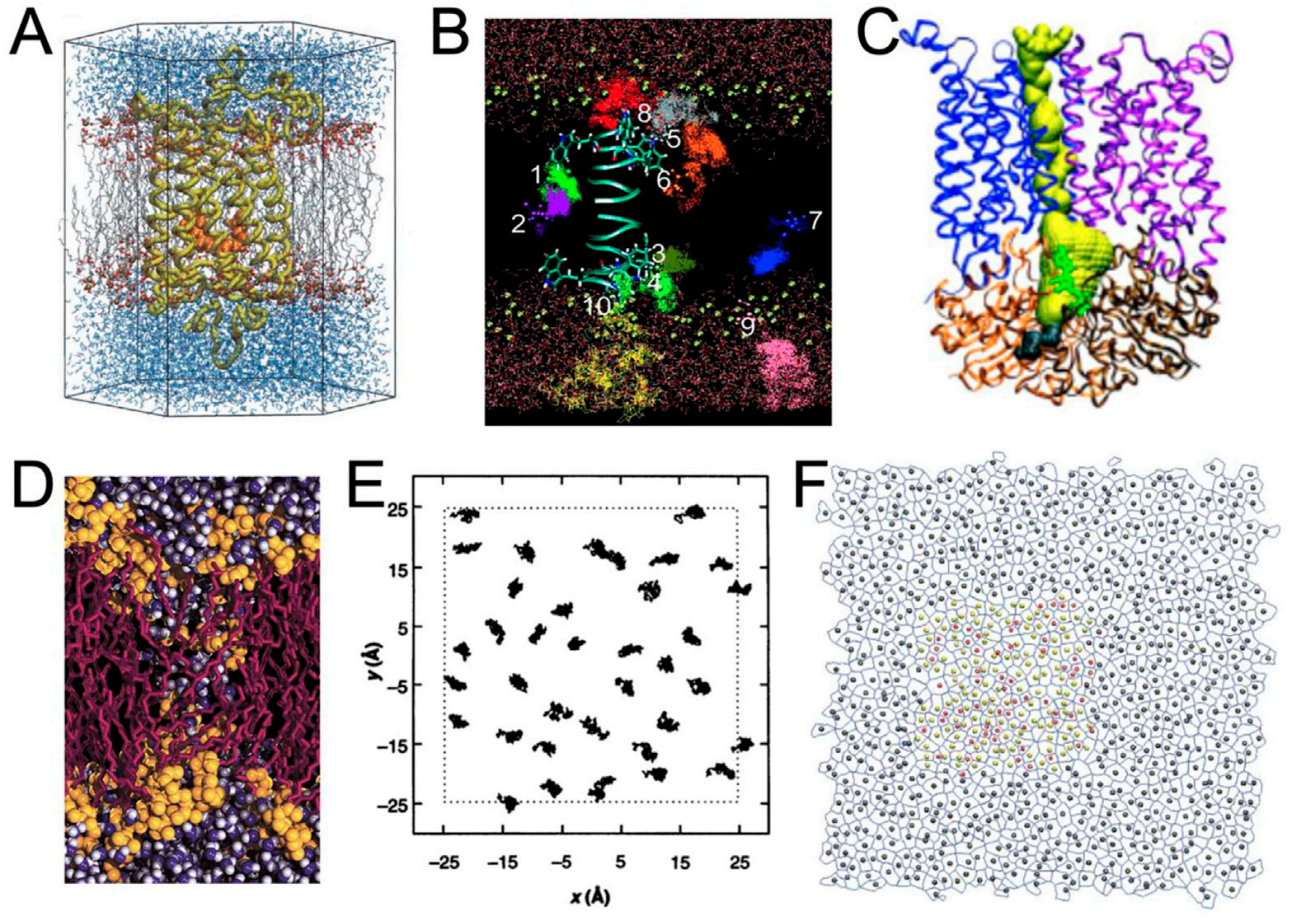
Examples of renderings performed during the period 1990–2005. **(A)** Rhodopsin (in yellow) with its ligand retinal (in orange) embedded in a POPC membrane (in gray and red) surrounded by water molecules (in blue) (Saam et al., 2002). *With the permission of the Theoretical and Computational Biophysics Group*, *University of Illinois Urbana-Champaign.* **(B)** Trajectories representation of the anesthetic halothane in a DMPC membrane (acyl chains not shown) along a gramicidin A peptide (Tang and Xu, 2002). **(C)** Ribbon representation of the BtuCD protein and representation of the pathway of vitamin B_12_ in yellow is HOLE program (Oloo and Tieleman, 2004). **(D)** Rendering of a transmembrane water pore (Marrink et al., 2001) using VMD in combination with an early version of the Tachyon ray tracing system developed by J Stone. *With the permission of* Pr *S.* (J)*. Marrink*. **(E)** 2D trajectories of lipids (Venable et al., 1993). **(F)** Example of rendering a Voronoi tessellation in combination with area per lipid analysis (Pandit et al., 2004).

It was then possible to study membrane proteins interacting with lipids and/or small molecules (Saam et al., 2002; Tang and Xu, 2002; Grossfield et al., 2006) (Figures 2A, B). The growing interest in ion channels and transporters has necessitated the visualization of pores in proteins. It was possible to render the protein surface to estimate the pore shape (Law et al., 2003; Sotomayor and Schulten, 2004), but this visualization was not optimal to also visualize important residues inside the proteins and dedicated tools, such as the program HOLE (Smart et al., 1996), were developed to both visualize and quantify pore shape (Doyle et al., 1998; Oloo and Tieleman, 2004) (Figure 2C). However, due to the increasing size of the model systems, it was necessary to select a portion of the model to zoom in and understand the interactions between specific residues (Gullingsrud et al., 2001; Sotomayor and Schulten, 2004).

The study of global biophysical changes involving numerous molecules was even more difficult to represent. One may cite lipid aggregation and fusion (Marrink et al., 2001; Marrink and Mark, 2003; Vries et al., 2004) (Figure 2D), molecular diffusion (Tang and Xu, 2002) (Figure 2B), or lipid packing (Pandit et al., 2004) (Figure 2F). In these cases, representation and analysis were often closely related (Figures 2B–E).

Compared to the previous decade, systems analysis (i.e., the use of diagrams) has not evolved as dramatically as molecular rendering. The main analyses were: z-density profile, order parameters, radial distribution functions, or membrane thickness calculations [see, e.g. (Falck et al., 2008)], often supplemented by 2D representations (Figures 2E, F).

After 2005, various methodological developments, often in parallel, have made it possible to model and visualize even more complex systems. So from here on we will present the last ∼15 years combined, focusing on issues of complexity versus advances in methodology/visualization.

## The development of CG force fields and the increase in system complexity required new visualization methods

**FIGURE 3:**
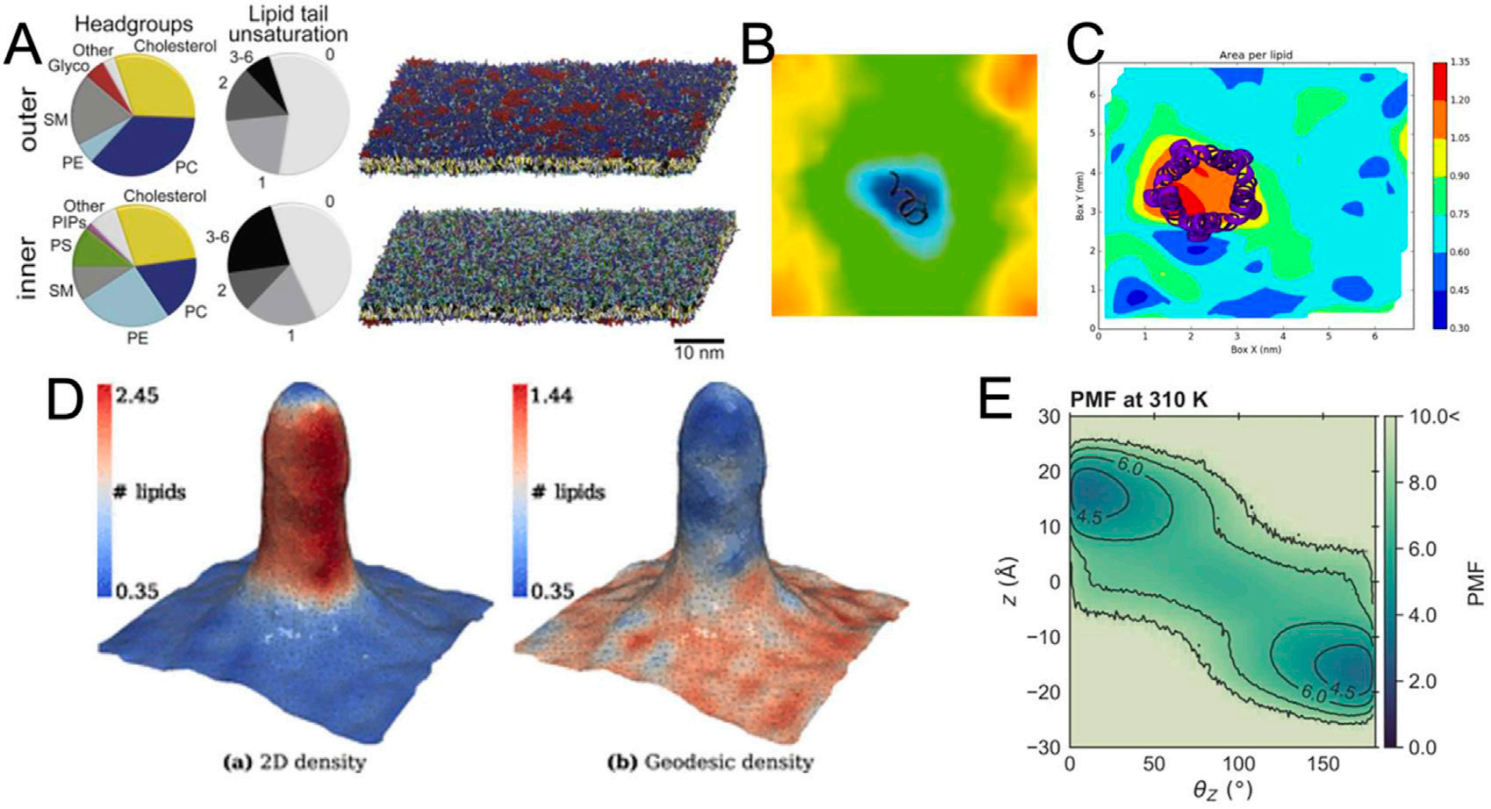
Exemplary visualizations of methods for the analysis of membrane bilayers **(A)** View of a complex membrane simulation showing the lipid composition (Ingólfsson et al., 2017). **(B)** Example for the visualization of the thickness of a bilayer around a peptide from GridMAT-MD (Allen et al., 2009) **(C)** Visualization of area per lipid from FATSLiM (Buchoux, 2016). **(D)** Extremely strong curvature visualized with MemSurfer (Bhatia et al., 2019). *Copyright American Chemical Society.* **(E)** Visualized PMF of lipid flipping made with LiPyphyilic (Smith and Lorenz, 2021).

A key development in this period has been the introduction of coarse-grained (CG) force fields. Early examples of these force fields focused on phospholipids (Shelley et al., 2001) and led to impressive early visualizations of self-assembling systems (Klein and Shinoda, 2008). Lipids were the focus of later CG force fields, such as the widely used Martini force field (Marrink et al., 2004; 2007). Shortly thereafter, the Martini force field was adapted to proteins (Bond and Sansom, 2006; Monticelli et al., 2008) and recently completely revised (Souza et al., 2021), leading to extensions of model systems well beyond lipids and proteins (Alessandri et al., 2021; Grünewald et al., 2022; Lutsyk et al., 2022). In parallel, other CG force fields were published, such as SIRAH (Darré et al., 2015; Machado and Pantano, 2016; Barrera et al., 2019; Machado et al., 2019) and ELBA (Orsi and Essex, 2011). These and other force fields have their own strengths and use cases and are discussed in more detail elsewhere (Ingólfsson et al., 2014a; Jin et al., 2022). With the development of CG force fields, new tools have been released to aid in the construction of complex membranes, such as the *insane* tool (Wassenaar et al., 2015) and CHARMM-GUI (Qi et al., 2015; Hsu et al., 2017) for constructing membrane systems. More recently, polyply (Grünewald et al., 2022) has been developed to easily create models for polymers, while TS2CG is used to create large and highly curved systems (Pezeshkian et al., 2020). For interested readers, more available tools are presented in the article (Javanainen and Martinez-Seara, 2016). This abundance of tools has opened the doors for modelling even larger and more complex membrane systems (Ingólfsson et al., 2014b; Ingólfsson et al., 2017) (Figure 3A). The resulting complexity presents a challenge for meaningful analysis and visualization of the data, where even the accurate representation of the range and identities of the lipids used requires careful consideration.

As membranes become more complex, not only are individual lipids and their dynamics visualized, but increasingly global membrane properties are represented, measured and quantified. For systems of intermediate size, there are several tools that can be used to visualize and analyze the membrane thickness (Figure 3B) or area per lipid (Figure 3C). Nevertheless, special tools have been developed to analyze and render complex features of membrane systems, including their curvature (Bhatia et al., 2019; Santos et al., 2020; Bruininks et al., 2021; Santos et al., 2022) (Figure 3D), or their volume and surface (Rozmanov et al., 2014). These programs not only provide detailed numerical information about the simulated system, but also strive to visualize the observed parameters in a clear and appealing manner (Figures 3B–E), which can be challenging for such complex systems. Since we are dealing with very large systems, an important goal of these programs is also to keep their analysis computationally efficient.

While the above methods tend to treat the membrane globally as a continuous plane, it is still necessary to scale down to individual lipid molecules, especially when considering molecular details. For example, lipid flip-flop rate and lipid-lipid interactions might be a necessary feature to analyze and visualize a complex membrane. These can be evaluated using, for example, the packages LiPyphyilic (Smith and Lorenz, 2021) (Figure 3E) or MOSAICS (Bernhardt and Faraldo-Gómez, 2022).

## Scaling up to organelles: Automation and simplification

As the ambitions of molecular modellers grow with computational power, there is naturally a desire to shift focus to ever larger, more complex regions of membranes (Chavent et al., 2016; Pezeshkian and Marrink, 2021; Gupta et al., 2022; Khalid et al., 2022). With these larger simulations comes a need for more elegant visualization methods. These must strike a good balance between being visually clear and appealing and conveying biological knowledge in an accurate and useful way. Furthermore, with such large systems, it is nearly impossible to capture both molecular details and potential effects at the mesoscale, forcing the researcher to juggle between different viewpoints. This can be a difficult task, requiring many hours of analysis and visualization to obtain meaningful representations and gain insight.

**FIGURE 4:**
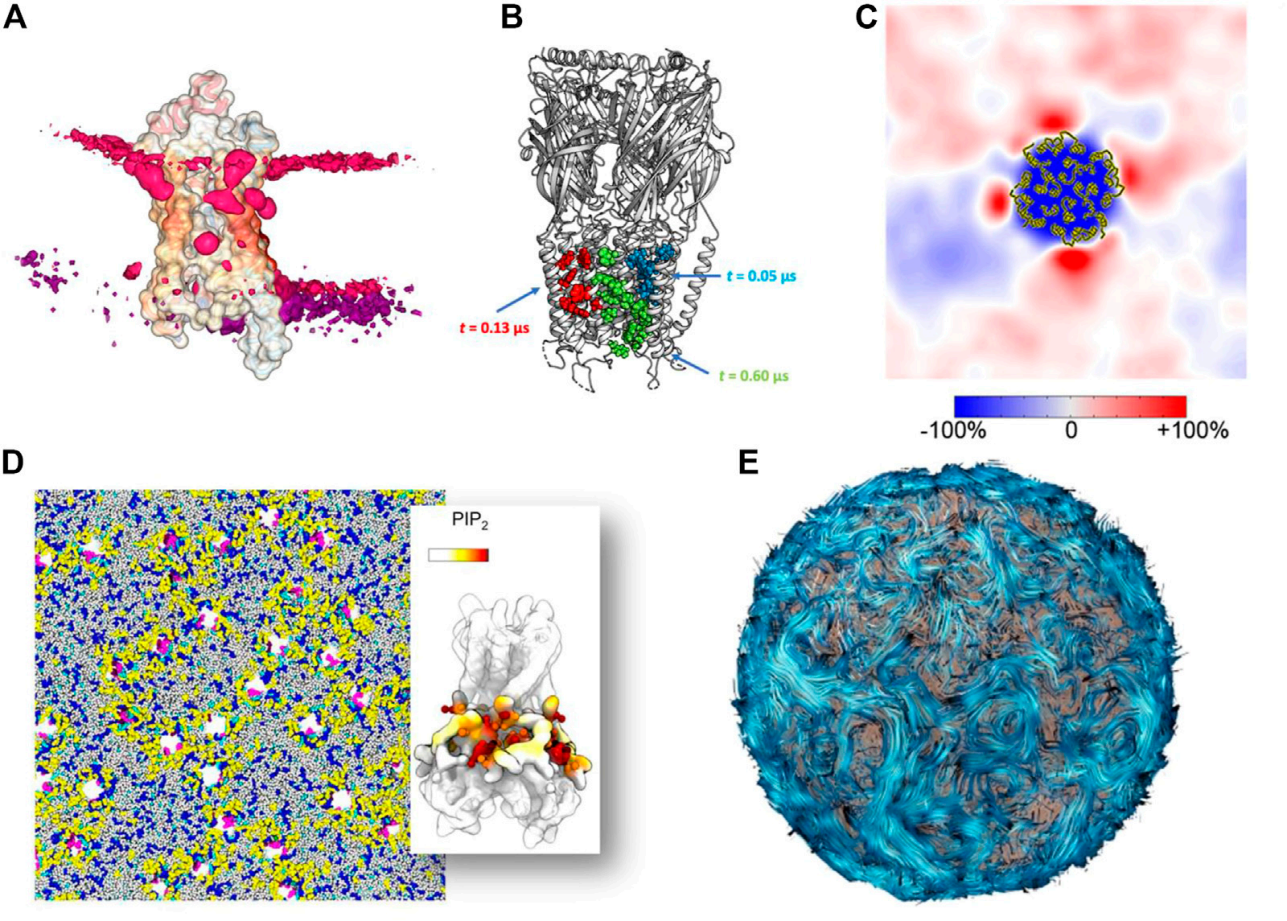
Methods for simplifying complex data into simple images **(A)** Lipid binding to a protein visualized with the ProLint web server (Sejdiu and Tieleman, 2021) **(B)** Lipid binding site analyzed and visualized with the PyLipID package (Song et al., 2022). **(C)** Lipid *fingerprint* around a membrane protein (Corradi et al., 2018). *Copyright American Chemical Society.* **(D)** Example of a complex membrane system with 144 receptors analyzed in detail at the molecular level (Duncan et al., 2020). **(E)** Example of the streamline method used to visualize lipid flows in a vesicle (Chavent et al., 2014).

At the molecular level, one solution is to apply a method that automates the evaluation of specific parameters. These programs are not only fast and reproducible, but allow the user to easily create impressive graphs and structural representations. For example, the tool ProLint (Sejdiu and Tieleman, 2021) is a powerful method for identifying protein-lipid interactions that provides a web server with interactive visualization (Figure 4A). PyLipID (Song et al., 2022) can also identifies and provides statistics on protein-lipid binding sites and automatically generates diagrams and scripts for molecular visualization (Figure 4B). These tools have been used to study large membrane systems, such as in a recent study of 144 Kir potassium channels in several complex membranes, which allowed us to analyze the preferential interactions of this protein with different lipids (Duncan et al., 2020) (Figure 4D). Another example is the study of a large number of membrane systems, showing the local enrichment of different lipid species around each membrane protein, so-called *fingerprints* (Corradi et al., 2018) (Figure 4C). The MemProtMD database also allows the visualization of protein-lipid interactions and getting statistics on a very large number of membrane proteins (Newport et al., 2018). Reducing the complexity of the system by creating simplified 2D images has been used to characterize the phase separation of lipids (Fowler et al., 2016) and lipid packing defects (Wildermuth et al., 2019). Aside from reducing the complexity of simulation data, the two-dimensional array format is likely to become increasingly important as it is integrated into machine learning (ML) workflows to bridge the different scales (Ingólfsson et al., 2022). This possibility is nicely illustrated by the example of lipid rafts, where such array representations have been used for domain recognition (Meinhardt and Schmid, 2019; Ho et al., 2022) and can indeed be used for supervised machine learning analysis (Canner et al., 2021). On a larger scale, understanding and visualizing the formation of protein networks has been used to decipher a growing number of membrane systems: from bacterial outer membrane (Chavent et al., 2018), chromatophore (Singharoy et al., 2019), to virus (Casalino et al., 2022) models. These models may require using new metaphors to render their biophysical properties. One such example is the streamline method (Chavent et al., 2014) (Figure 4E), which visualizes lipid dynamics over large areas in a clear and visually appealing manner. A variant was used to represent a lipid nanoreactor embedded in water. In this case, the water flow, modelled by a Lattice-Boltzmann approach, was represented by field lines, while the vesicle was explicitly represented by Van der Waals spheres (Brandner et al., 2019). Therefore, data simplification for very large models will continue to be important for analysis and visualization.

## Shedding new light on large and crowded systems

**FIGURE 5:**
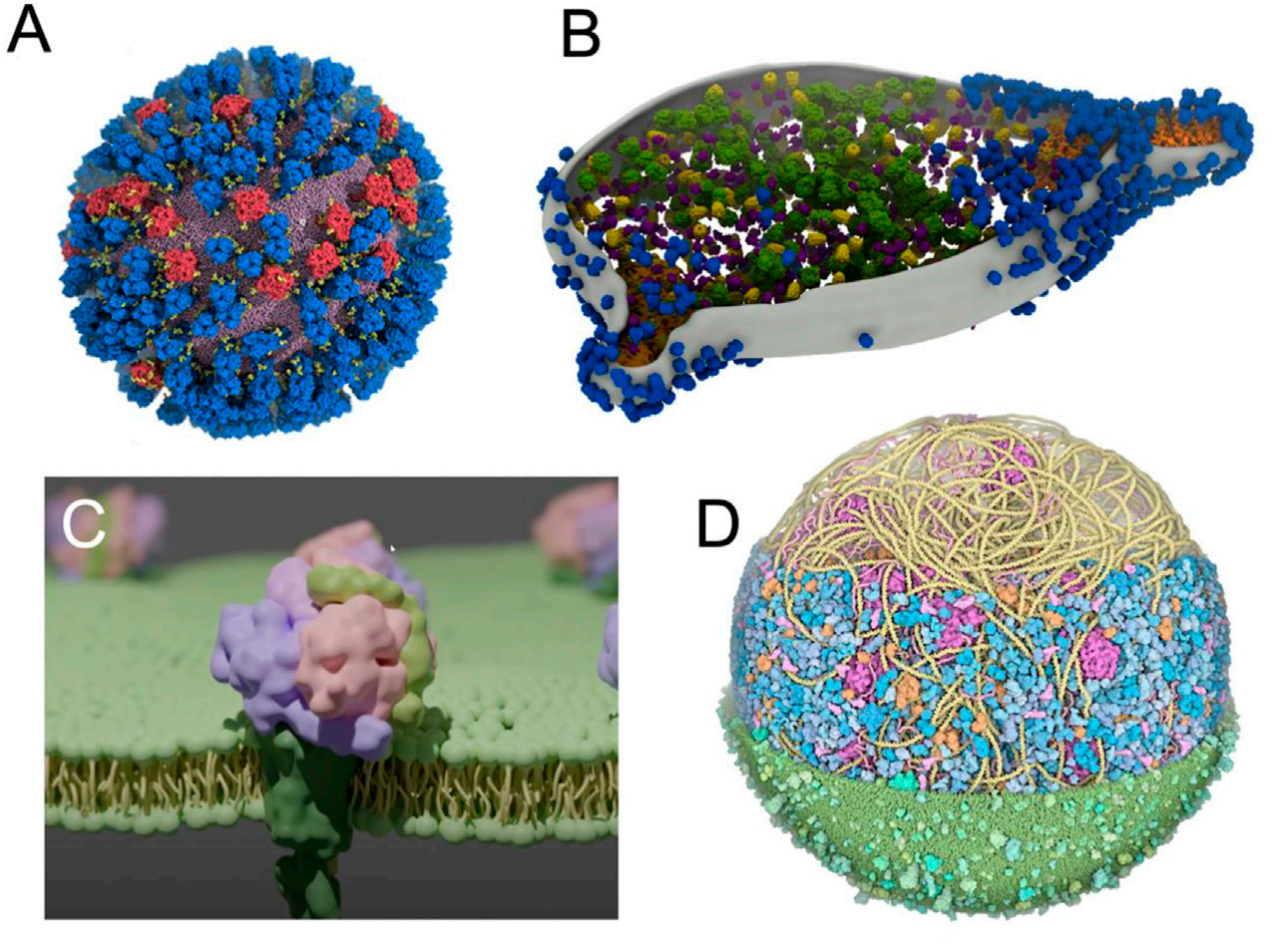
Using advanced computer graphics to make visually striking images **(A)** View of an influenza A virus rendered with VMD (Casalino et al., 2022). *Copyright American Chemical Society.* **(B)** *M*. *genitalium* cell visualised with experimental data using ArtiaX (Ermel et al., 2022) **(C)** A membrane protein embedded in a membrane created with Blender’s MolecularNodes plugin (Johnston, 2022) **(D)** An image of a complete *mycoplasma* cell created with CellPAINT (Maritan et al., 2022).

Increasing efforts have been made to improve the visual quality of snapshots and molecular poses from large molecular simulations using software such as VMD (Jefferies and Khalid, 2020; Vermaas et al., 2021; Casalino et al., 2022) (Figure 5A), which is constantly evolving and becoming more powerful over time (Stone et al., 2013; Stone et al., 2016; Stone, 2019). In parallel, other visualization methods are increasingly being used and have recently been updated, such as the new version of the Chimera software, called ChimeraX (Goddard et al., 2018b), including extensions such as ArtiaX (Ermel et al., 2022) (Figure 5B), both of which set new standards in terms of visual representation of membranes and membrane proteins. Similarly, tools such as Blender3D (Community, 2018) MolecularNodes plugin allow for the rapid rendering of very complex systems that produce impressive results (Johnston, 2022) (Figure 5C). Due to its optimized rendering algorithms and, user-friendly and exhaustive user interface, Blender3D program allowed efficiently managing the rendering of an entire minimal cell model (Stevens et al., 2023). New molecular renders are also emerging, directly integrating advanced lighting and rendering (Chavent et al., 2011; Lv et al., 2013; Maria et al., 2022).

When the simulated environments become even more crowded (Yu et al., 2016; Bülow et al., 2019; Bari et al., 2023), more artistically inclined methods can be used, such as in the work of Dr. David Goodsell (Goodsell et al., 2018). This visualization was then stratified into a set of tools, such as CellPAINT (Gardner et al., 2021), that combines visualization and modelling to create models such as a *mycoplasma* cell (Maritan et al., 2022) (Figure 5D).

## Depicting large dynamical motions of membrane systems

Beyond the examples presented here, different works have highlighted the importance of studying on larger scales the motions of lipids (Baoukina et al., 2017; Hsu et al., 2017; Vögele et al., 2018) and membrane proteins (Periole et al., 2012; Arnarez et al., 2016; Song et al., 2021), as well as broader membrane deformations (Simunovic et al., 2013; West et al., 2016; Siggel et al., 2021). To provide just one example in which such large dynamic motions are essential, we should mention the processes of membrane invagination, which have been studied at different levels of modeling (Pezeshkian et al., 2016; Pezeshkian et al., 2019). These phenomena are difficult to represent by static pictures. A straightforward solution is to propose movies in supplementary material see e.g. (Simunovic et al., 2013; Chavent et al., 2018; Casalino et al., 2022; Schaefer and Hummer, 2022) but this rendering is limited to the camera view point.

Recent works to render large molecular motions may be of help here. Adding additional motion information on top of the systems such as arrows (Bryden and Gleicher, 2012) may help understanding the overall directions of movements. Coloring molecular path in function of predefined criteria could highlight specific molecular motions as done recently for water molecules trajectories (Vad et al., 2017). Another solution can be to couple 3D rendering with 2D interactive maps of diffusion, interactions and clustering to gain insights into the correlation of protein-lipid and protein-protein interactions (Alharbi et al., 2019).

## New solutions to old problems

Thus**,** throughout the history of membrane modelling, increasing the complexity and the size of the systems have led to visualization and analysis breakthroughs. Nevertheless, the dawn of exascale computing will lead to a complete paradigm shift in terms of model size and complexity, as we are starting to witness (Dommer et al., 2021). The modelling community needs to be prepared to handle such systems and will need some help from other communities. One solution is to look at recent computer scientist’s approaches to better visually understand, simplify, and/or abstract complex membrane systems (Martinez et al., 2019). Another help could come from designers (Sommer et al., 2022; Spalvieri et al., 2022). These different experts could also contribute their unique perspectives and skills to find new and innovative ways to represent and potentially abstract these complex systems (Viola and Isenberg, 2017).

It is still a challenge to capture the intricacies of complex membrane systems using only 2D images. We have used several examples to show the importance of switching between magnified molecular details and downscaled views. Perhaps it will soon be time to leave the flat 2D image behind and turn to the 3D environment instead. This next step will be possible with the advances in virtual reality (VR) and augmented reality (AR) (Goddard et al., 2018a; O’Connor et al., 2019; Baaden, 2022; Kut’ak et al., 2023).

To date, not many groups have documented experiments in the literature using AR and VR to visualize membrane systems. This scarcity may be because these approaches are often used as a tool in a research project, but are not necessarily the actual focus of the work. It is very likely that many more immersive experiments have been conducted than a literature search would reveal, as membrane objects lend themselves very well to such visualizations (Martinez et al., 2020b; Baaden, 2022). As lipid diffusion and currents can be naturally translated into field lines, it is already possible with VR to visualize such type of rendering properties (Laureanti et al., 2020). With the increasing adoption of VR and AR technology in the scientific community, it is likely that these approaches will be extensively explored for membrane visualization in the near future.

A final step will be to integrate the visualization of these models with the visualization of biological membranes obtained by experimental methods (Baaden, 2019), to blur the boundaries between theory and experiment. Recent works are already moving in this direction (Thornburg et al., 2022; Stevens et al., 2023). Moreover, with the COVID pandemic, we have seen an unprecedented use of molecular imagery in non-technical forums such as the news^1^. This trend highlights the power that these visualizations can have to capture the public’s imagination and emphasizes the need to share such visual experiences broadly (Martinez and Baaden, 2021; Kampfrath et al., 2022).

## Data Availability

The original contributions presented in the study are included in the article/supplementary material, further inquiries can be directed to the corresponding author.

## Footnotes

1
https://www.nytimes.com/interactive/2021/12/01/science/coronavirus-aerosol-simulation.html
